# East Line Medical Society of Texas

**Published:** 1888-10

**Authors:** 


THE EAST LINE MEDICAL ASSOCIATION OF TEXAS met in Greenville on the 2nd inst. Drs. Musick, Westbrook, Patterson, Radford, M. S. B. and O. Smith and Crutcher read editorial papers. Dr. A. H. Evans is Secretary.

